# Comparative study on corneal epithelium healing: effects of crosslinked hyaluronic acid and amniotic membrane extract eye drops in rats

**DOI:** 10.3389/fvets.2024.1415658

**Published:** 2024-07-24

**Authors:** Lenara Gonçalves e Souza, Matheus Vilardo Lóes Moreira, Claudia Sayuri Saçaki, Eduardo Perlmann, Thacyana Beatriz Guimarães Lopes, Enio Ferreira, Juan Carlos Duque Moreno, Fabiano Montiani-Ferreira

**Affiliations:** ^1^Veterinary Medicine, Department, Rua dos Funcionarios, Graduate School of Veterinary Sciences, Federal University of Parana, Curitiba, Brazil; ^2^Laboratory of Veterinary Pathology, MVL Patologia Veterinária, Belo Horizonte, Brazil; ^3^Graduate School Department, Pelé Pequeno Príncipe Research Institute, Curitiba, Brazil; ^4^Veterinary Ophthalmology Department, Petcare Hospital, São Paulo, Brazil; ^5^Veterinary Ophthalmology Department, Vetmasters Clinic, São Paulo, Brazil; ^6^Department of General Pathology, Institute of Biological Sciences, Federal University of Minas Gerais, Belo Horizonte, Brazil

**Keywords:** cross-linked hyaluronic acid hydrogel, amniotic membrane extract, eye drops, epithelial healing, rat, histopathology

## Abstract

**Introduction:**

Corneal ulcers are common lesions in both human and veterinary medicine. However, only a few studies have evaluated the efficacy of cross-linked hyaluronic acid (X-HA) eye drops on corneal wound healing. To our knowledge, this is the first study to demonstrate and compare the efficacy of amniotic membrane extract eye drops (AMEED) and X-HA for corneal wound healing in rats.

**Material and methods:**

A total of 15 male Wistar rats (30 eyes) were used in this study. Then, 10 eyes were treated with X-HA, AMEED, or 0.9% saline. After general and topical anesthesia, a superficial corneal ulcer was created using a corneal trephine. The defect was further polished with a diamond burr. Three groups of 10 eyes each were treated with either one drop of 0.75% X-HA or AMEED or 0.9% saline (control), administered every 12 h for a duration of 72 h. The median epithelial defect area (MEDA), expressed as a percentage of the total corneal surface, was measured at 0, 12, 24, 36, 48, and 72 h. Re-epithelization time scores were also evaluated. The Kruskal–Wallis test was used to compare median times for re-epithelization and histopathologic scores between groups, while the Friedman test (for paired data) was employed to compare results from the serial analysis of MEDA and vascularization scores between groups.

**Results:**

MEDA was not significantly different between X-HA and AMEED. However, MEDA was significantly smaller in the X-HA group compared to the control group at 36 h (2.73 interquartile range (IQR) 5.52% x 9.95 IQR 9.10%, P=0.024) and 48 h (0.00 IQR 0.26% x 6.30 IQR 8.54%, P=0.030). The overall time for re-epithelization was significantly lower in the X-HA group (3.00 IQR 3.00) compared to the AMEED (6.5 IQR 3.00) and control (7.00 IQR 1.00) groups (P=0.035). Vascularization, hydropic degeneration, and epithelial-stromal separation were significantly less observed in samples in the X-HA-treated compared to samples in the AMEED- and saline-treated groups. Significantly more corneal epithelium cells were labeled for caspase3 in samples from the AMEED- and saline-treated groups compared to those from the X-HA-treated group.

**Discussion:**

Topical X-HA has been shown to accelerate corneal epithelial healing. AMEED did not decrease corneal re-epithelialization time. X-HA may also potentially be used as an adjunct therapy for treating corneal ulcers in clinical situations.

## 1 Introduction

Corneal ulcers possess many etiologies and presentations, often representing a clinical challenge. Epithelial sliding and anterior stromal replacement are the main events in the process of superficial corneal ulcer healing. Anterior stromal replacement requires the synthesis and cross-linking of collagen, proteoglycan synthesis, and gradual wound remodeling (1). Delayed corneal epithelial healing may lead to subsequent corneal infections with further complications, such as corneal scarring, thinning, ulceration, and even perforation. The initial topical treatment is an important stage in the further medical management of corneal ulcers. Recently, two newer active ingredients for topical use, which show evidence of improving corneal healing, became part of the medical treatment arsenal: cross-linked hyaluronic acid (X-HA) and amniotic membrane extract (AME). There is limited evidence indicating that X-HA hydrogel provides some benefit during healing by accelerating the time to corneal wound closure, especially when compared to a non-cross-linked HA solution in companion animals (2). Amniotic membrane (AM) has been extensively used to accelerate corneal wound healing (3–7). In theory, AME contains many properties similar to cryopreserved AM. AME is often produced for use in other fields of medicine, while amniotic membrane extract eye drops (AMEED) are defined as AMEs designated specifically for topical ophthalmic use (8). Understanding corneal healing and the potential therapeutic interventions to optimize this natural process is crucial. This study aimed to investigate and compare the effects of cross-linked hyaluronic acid eye drops and a commercially available AMEED on corneal wound healing in rats. A control group of treated eyes in which topical saline was used at the same frequency was also included for comparison purposes. Epithelization time, changes in the experimentally created ulcer area, and healing quality using histopathology were analyzed through serial clinical images and histopathologic analyses.

## 2 Materials and methods

### 2.1 Ethics committee

The present animal study was approved and reviewed by the Ethics Committee of Pelé Pequeno Príncipe Research Institute under certificate number 064-2022. All procedures performed in the study were in accordance with the Association for Research in Vision and Ophthalmology (ARVO) Statement for the Use of Animals in Ophthalmic Vision and Research.

### 2.2 Type of study

Experimental investigation.

### 2.3 Animal model and conditions

A total of 15 male Wistar rats (30 eyes) weighing 200–230 g were selected for this study. The rats were provided with *ad libitum* access to species-specific food and water. They were housed in an environment with a 12-h light/dark cycle (light switched on at 8:00 AM and switched off at 8:00 PM) and maintained under controlled temperature conditions with a mean temperature of 22°C (±2°C). During the experimental procedure (72 h), each rat was housed individually (isolated) in smaller propylene cages measuring 435 mm (length) x 290 mm (width) x 160 mm (height) with sawdust bedding.

### 2.4 Experimental design

A total of 30 eyes of 15 rats were treated with either X-HA, AMEED, or saline (involving 10 eyes per treatment group). The X-HA (cross-linked hyaluronic acid) 0.75% eye drops (Oculenis Biohance^®^, SentrX, Salt Lake City, UT, USA), AMEED (amniotic membrane extract eye drops, EyeQ^®^ Amniotic Eye Drops, Vetrix^®^, Cumming, GA, USA), or 0.9% saline were assigned to each eye using random number generator software (https://www.random.org/). The randomization was aimed at eliminating bias and ensuring unbiased data collection and analysis.

### 2.5 Experimental corneal lesion induction

Pre-operative systemic anti-inflammatory and analgesic medication: Meloxicam (2 mg/kg) (Maxicam 1 mg/ml, Ourofino Animal Health, Cravinhos, SP, Brazil) was administered subcutaneously starting 2 h prior to the surgical procedure continued for up to 48 h after injury for pain control, as previously described by Genova et al. (9). General anesthesia was administered to the rats using inhalational anesthesia with isoflurane (3% induction followed by 2% maintenance, with oxygen) at the rate of 1.5 L/min. The corneal area was then topically anesthetized using 1% tetracaine hydrochloride eye drops. The microsurgery was performed under an operating microscope (DF Vasconcellos, Valença, RJ, Brazil). Each eye was previously prepared aseptically with a 0.25% diluted povidone-iodine solution prior to the procedure. Based on the methodology described by Portela (10), a superficial lamellar keratectomy was performed using a trephine with a diameter of 3.0 mm (Disposable Biopsy Punch^®^, Integra, Princeton, NJ, USA). The trephine was gently rotated to demarcate the precise margin required. A superficial lamellar keratectomy was then finalized using a 2.0-mm crescent knife (Diamatrix, The Woodlands, TX, USA). The bed of the central corneal epithelial defect was further polished with a fine diamond burr (Alger Brush II, Alger Company, Lago Vista, TX) using multiple, gentle, circular movements, taking special care to avoid making the lesion wider or inducing irregular topography by either pressing too firmly or remaining in one focal area too long, creating a standardized pre-established corneal epithelial defect.

### 2.6 Topical treatment protocol

Following the induction of corneal injury, all three groups received one drop (0.05 mL) of the respective eye drops (X-HA, AMEED, and saline) every 12 h, specifically targeting the injured eye. This treatment regimen was adhered to for the entire duration of the experiment (72 h). Steroids and antibiotics were not used because they could potentially interfere with the corneal healing process.

### 2.7 Evaluation of corneal healing

To monitor the progress of corneal re-epithelialization, the animals were assessed at predetermined time points: 0, 12, 24, 36, 48, and 72 h. The eye surface was examined using a slit lamp (HSL-01 Digital Handheld Slit Lamp, Hyperion, MicroClear Medical Instruments, Suzhou, China), stained with 1% sodium fluorescein, and photographed with the same equipment using a cobalt blue light filter. To maintain uniformity, the distance between the slit lamp and the eye surface was kept constant using a 5-cm spacer. Following the protocol of Portela et al. (10), lesions were analyzed for only up to 72 h. If, after 72 h, a given lesion was not fully re-epithelized, it was labeled as “incomplete epithelization.” The following re-epithelization scores were assigned based on the time taken for complete re-epithelization time: (1) 12 h; (2) 24 h; (3) 36 h; (4) 48 h; (5) 72 h; and (6) incomplete re-epithelization in 72 h. The fluorescein-stained area of the epithelial defect area was proportionally calculated and expressed individually as the percentage of the ulcerated area relative to the total corneal surface area at each time point, referred to as the median epithelial defect area (MEDA). In addition, clinical vascularization scores (0–3) were analyzed using a modified Hackett-McDonald scoring system (11–13). These analyses were conducted using commercially available image analysis software (Image-Pro Plus^®^V7; Media Cybernetics, Inc.; Rockville, MD, USA).

### 2.8 Euthanasia procedure

After 72 h of treatment, the animals were humanely euthanized. Intraperitoneal injection of 60 mg/kg ketamine (Ketamin S, Cristália, São Paulo, Brazil) and 10 mg/kg xylazine (Sedomin 10%, König Brasil S.A., São Paulo, Brazil) was administered, along with 0.03 mg/kg fentanyl citrate (Fentanest; Cristália, São Paulo, SP, Brazil) for analgesia to minimize any potential stress, pain, or discomfort. Once complete anesthesia was achieved, a lethal dose containing ketamine (180 mg/kg) and xylazine (30 mg/kg) was administered via an intraventricular injection. The animals' weights were measured before any procedures to ensure accurate dosage calculations.

### 2.9 Histopathologic and immunohistochemical analysis

For the histopathologic analysis, the eyes were enucleated and fixed in 10% neutral buffered formalin. The tissues were then dehydrated using a graded series of alcohol and embedded in paraffin. Subsequently, 5-μm paraffin sections were obtained, with the globes being cut at the central corneal lesion and stained with hematoxylin and eosin for the microscopic evaluation of the corneal tissue. Histopathology features were analyzed with regard to cellularity, the disposition of collagen fibers, and inflammatory infiltration. Three random high-power fields in the center of the corneas (at the recently epithelized site or immediately adjacent to the healing lesion area) were analyzed and scored on a scale of 0 to 4 (0 being absent; 1–4 indicating increasing severity, 4 being most severe). Immunohistochemical staining was performed using the peroxidase reaction method with a polymerized secondary antibody and the chromogen 3′3-diaminobenzidine (Novolink Polymer Detection System; Leica Biosystems, Newcastle upon Tyne, UK). Antigen retrieval was performed by incubation in citrate buffer (pH 6.0) in a water bath for 20 min at 98°C or Pascal^®^ Pressure Cooker (Dako Cytomation, Glostrup, DNK) for 2 min at 125°C. The following antibodies were used: Caspase-3Cliv (Clone E87, 1:200, Millipore^®^, Burlington; Massachusetts) and cytokeratin with a high molecular weight (Clone 34βE12, 1:200, Dako^®^, Santa Clara, CA, USA). The incubation time for the primary antibody was set at 16 h. The tissue slides were counterstained with hematoxylin for 10 s. A total of 27 sections (9 eyes from each group) stained with immunohistochemistry were evaluated and compared qualitatively (cytokeratin) and quantitatively (caspase 3). The latter was determined by counting the number of cells labeled for caspase 3 in 10 high-power fields (40x magnification).

### 2.10 Statistical analysis

The D'Agostino-Pearson test was conducted to assess the normal distribution of the data. The results indicated a deviation from the normal distribution. The following non-parametric tests were used: (1) The Kruskal–Wallis test was used to compare the median number of cells labeled for caspase 3, histopathologic scores, and median time for re-epithelization scores; (2) the Friedman test (for paired data) was employed to compare results from the serial analysis of the median percentage of ulcerated area between groups and vascularization scores. *P*-values of < 0.05 were considered statistically significant. Since the resulting data were non-normally distributed, the median and interquartile range (IQR) were used to demonstrate all descriptive results. The sample size was determined through the consideration of the percentage of ulcerated area in relation to the total corneal surface, using a type I error of 0.05 and at least 80% power (type II error of 0.02), with an estimated difference of 23.2%. For pairwise comparisons, the standard deviation of sample 1 was set at 13.63%, and the standard deviation of sample 2 was 12.08%. The ratio of sample sizes between groups is 1. The resulting number of cases required was greater than or equal to six eyes per group. For all these analyses, MedCalc^®^ Statistical Software version 20.027 (MedCalc Software Ltd, Ostend, Belgium) was used.

## 3 Results

**Figure 1:**
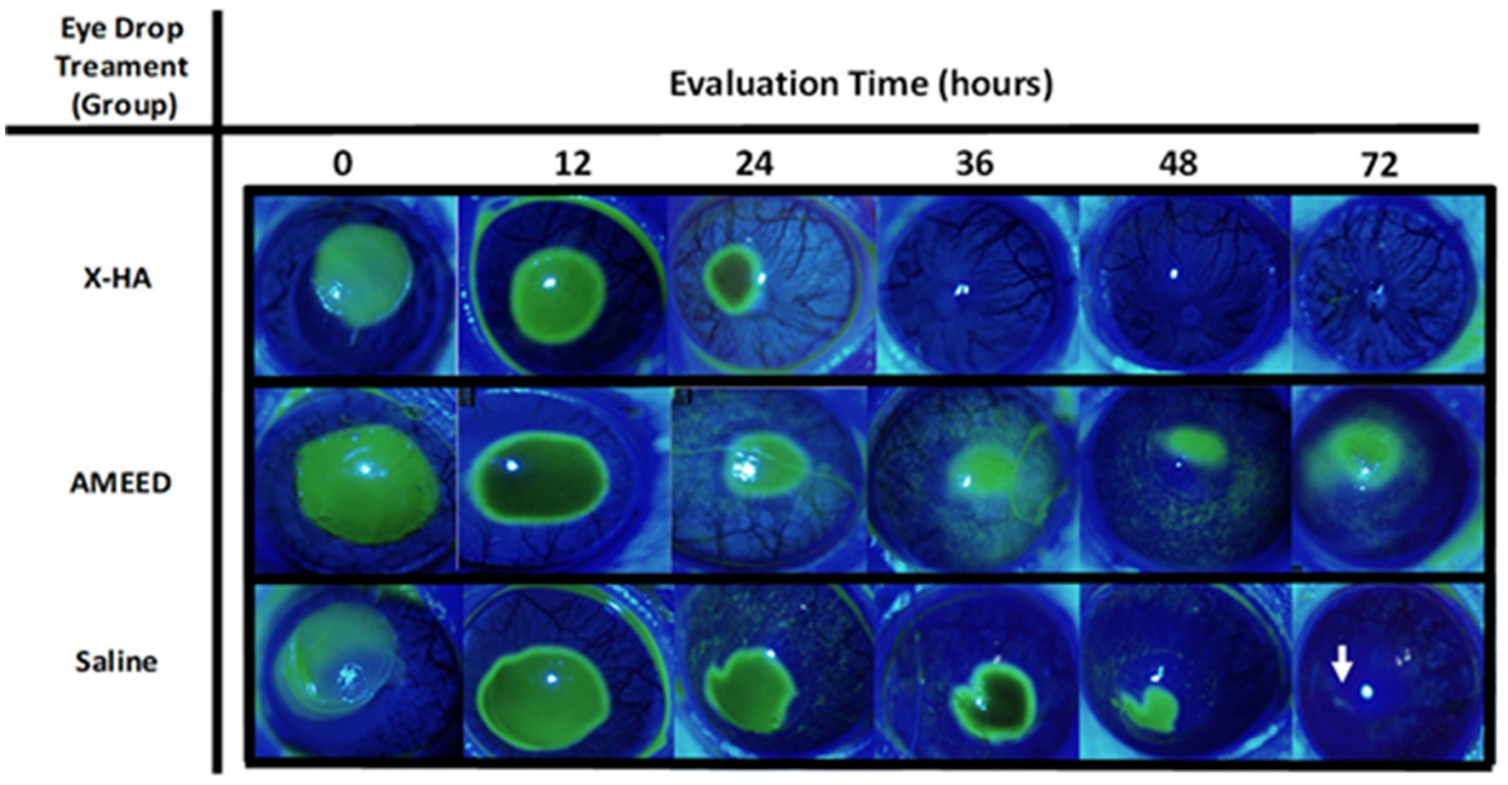
Representative ocular surface photographs of individual animals from each eye drop in the group X-HA (animal # 10, ID 18), AMEED (animal #4, ID 04), and saline (animal #5, ID 05) at each evaluated time point. Note that re-epithelization occurs at the following time points: Animal # 10, ID 18, treated with X-HA at 36 h (re-epithelization time score of 4) and Animal #4, ID 04, treated with AMEED, showed incomplete re-epithelization (IR) at 72 h (re-epithelization time score of 7). Note the presence of loose epithelium margins surrounding the ulcer and fluorescein stain undermining its border at 72 h; Animal #5, ID 05 also showed incomplete re-epithelization. Note a small fluorescein-positive lesion near the center of the cornea (white arrow) at 72 h (re-epithelization time score of 7).

**Figure 2:**
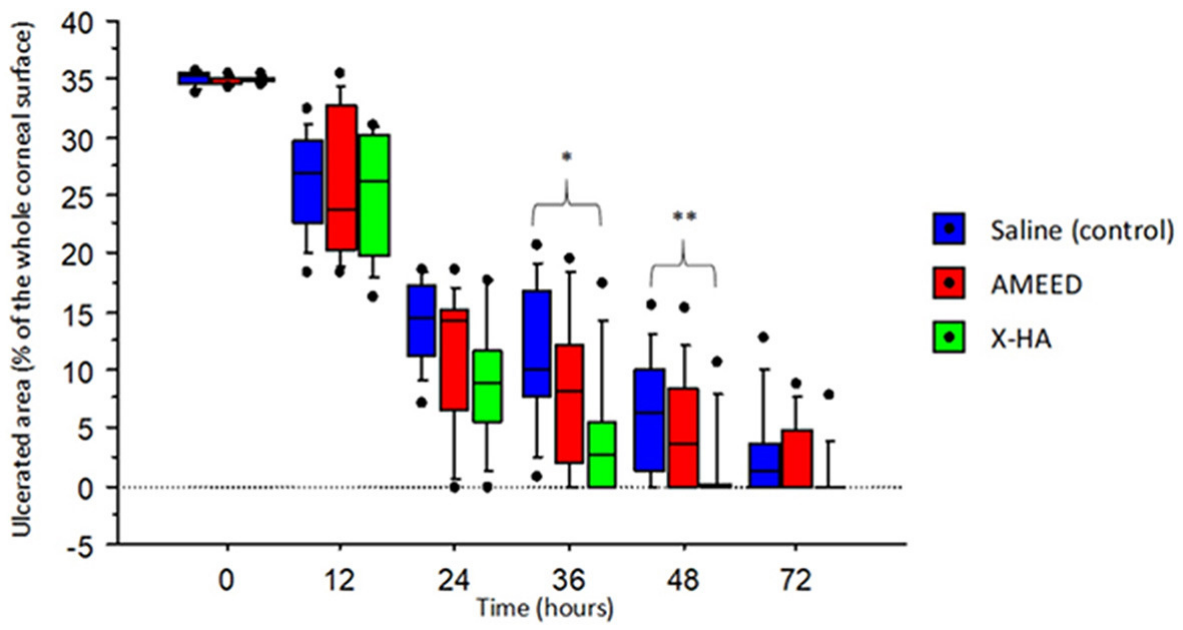
A box plot graph depicting the median, minimum, maximum, IQR, and outliers of the ulcerated percentage of the total corneal surface area, or median epithelial defect area (MEDA), at each different time point evaluated. Note the consistently smaller areas observed in the X-HA-treated eyes, starting at 24 h of treatment. *The difference between X-HA and saline (control) was significant (*P* = 0.024) at 36h. **The difference between X-HA and saline (control) was significant (*P* = 0.030) at 48h.

**Figure 3:**
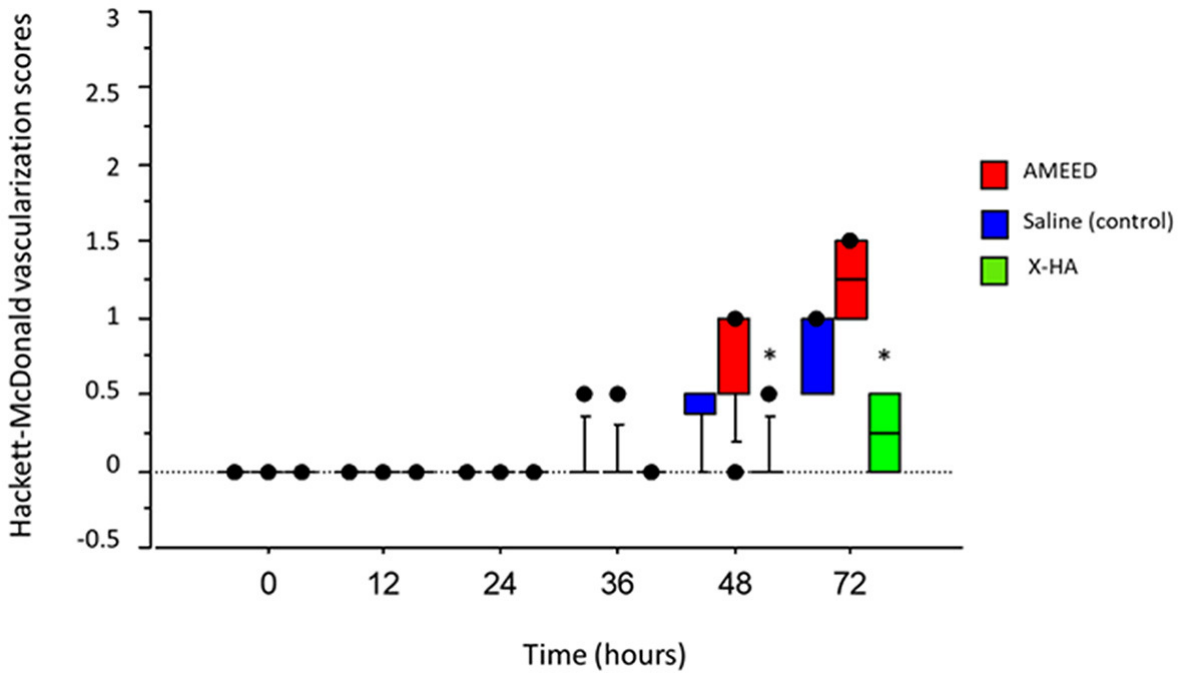
A box plot graph depicting detailed descriptive statistics (median, minimum, maximum, I, QR, and outliers) of the vascularization scores for each time point evaluated for each treatment group. *By 48h, a significantly lower median vascularization score was observed in the X-HA group compared to the AMEED, P=0.01, and saline, P=0.008, groups. By 72h, a significantly lower median vascularization score was observed in the X-HA group compared to the AMEED, P=0.012, and saline, P=0.005, groups.

A qualitative slit-lamp examination revealed slight corneal opacities in the area of the experimental ulcers that were quite similar across all groups during the first 36 h. Nevertheless, in the late stages of the healing process, loose corneal epithelium surrounding the ulcer margins (in which the fluorescein stain would undermine its border) was observed in four eyes of the AMEED group (4/10, in 72 h) and two eyes of the saline group (2/10, in 48 h). Moreover, corneas in these two groups also lost surface smoothness in the central area more markedly compared to eyes in the X-HA-treated group in the late stages of the healing process. Animal numbers, which refers to each animal's identification inside the laboratory, the treated eye, time for re-epithelization, respective scores for each animal, and the treatment groups allocated are shown in Table 1. The type of topical drug used (treatment) significantly influenced re-epithelization scores (*P* = 0.035). An overall significantly lower time for re-epithelization was observed in the X-HA-treated group (3.00 IQR 3.00) compared to the AMEED- (6.50 IQR 3.00) and saline-treated (7.00 IQR 1.00) groups. The inferential statistics results and a comparison of the re-epithelization scores are summarized in Table 2. Within each treatment group, the median percentage of ulcerated area in relation to the total corneal surface significantly decreased at each evaluated time for all treatments (*P* < 0.05), with exceptions at the following time points: 24 h vs. 36 h for saline (14.50 IQR 6.10% vs. 9.95 IQR 9.10%, *P* = 0.15); 24 h vs. 36 h for AMEED (14.42 IQR 7.56% vs. 8.12 IQR 9.99%, *P* = 0.12); and 48 h vs. 72 h for X-HA (0.00 IQR 0.26% vs. 0.00 IQR 0.00%, P=0.70). Representative ocular surface photographs of individual animals from each eye drop group at each evaluated time point are shown in Figure 1. The X-HA group showed consistently smaller ulcerated areas from 24 h to 72 h of treatment. Compared to the saline group (control), significantly lower medians of the percentage of ulcerated area were observed in the X-HA group at the following time points: at 36 h: 2.73 (IQR: 5.52%) X-HA vs. 9.95 (IQR 9.10%) saline (*P* = 0.024) and at 48 h: 0.00 (IQR 0.26%) X-HA vs. 6.30 (IQR 8.54%) (*P* = 0.030). Table 3 shows overall descriptive results pertaining to the evolution of the percentage of ulcerated area in relation to the total corneal surface for each treatment group. Figure 2 shows a graph depicting inferential statistics results and detailed descriptive statistics (median, minimum, maximum, IQR, and outliers) of the ulcerated percentage of the total corneal surface area at each evaluated different time point. Discrete vascularization was observed between 36 and 48 h (Figure 3). An overall significant difference in vascularization was observed in AMEED- and saline-treated corneas compared to X-HA-treated ones (*P* = 0.0085). At 48 h, a significantly lower median vascularization score was observed in the X-HA group (0.00 IQR 0.00) compared to the AMEED (0.5 IQR 0.12, *P* = 0.01) and saline (0.50 IQR 0.50, *P* = 0.008) groups. At 72 h, a significantly lower median vascularization score was observed in the X-HA group (0.25 IQR 0.50) compared to the AMEED (1.00 IQR 0.50, *P* = 0.012) and saline (1.25 IQR 0.50, *P* = 0.005) groups (Figure 3).

**Table 1:** Identification of the animals studied, eye treatment identification, allocation of treatment (eye drop used), time for re-epithelization, and respective re-epithelization scores.

**Animal number**	**Animal lab ID**	**Eye**	**Eye drop used**	**Time for re-epithelization (h)**	**Re-epithelization time (score)**
1	35	OD	X-HA	36	3
1	35	OS	Saline	48	4
2	41	OD	Saline	IR	7
2	41	OS	AMEED	72	6
3	42	OD	X-HA	36	3
3	42	OS	Saline	IR	7
4	04	OD	AMEED	IR	7
4	04	OS	Saline	IR	7
5	05	OD	Saline	48	4
5	05	OS	X-HA	IR	7
6	06	OD	Saline	IR	7
6	06	OS	X-HA	IR	7
7	07	OD	AMEED	IR	7
7	07	OS	Saline	72	6
8	09	OD	Saline	72	7
8	09	OS	AMEED	IR	7
9	17	OD	AMEED	IR	7
9	17	OS	Saline	72	6
10	18	OD	X-HA	36	3
10	18	OS	Saline	IR	7
11	E1	OD	X-HA	24	2
11	E1	OS	AMEED	IR	7
12	E2	OD	AMEED	72	6
12	E2	OS	X-HA	72	6
13	E3	OD	AMEED	48	4
13	E3	OS	X-HA	48	4
14	E4	OD	X-HA	36	3
14	E4	OS	AMEED	36	3
15	E5	OD	AMEED	36	3
15	E5	OS	X-HA	36	3

OD, oculus dexter, right eye; OS, oculus sinister, left eye.

**Table 2:** Descriptive and inferential statistics results, depicting minimum, maximum, 25th percentile, median, 75th percentile, maximum time for re-epithelization, and *p*-values of the inferential comparison (Kruskal–Wallis test) of the scores for the eye drop group investigated.

**Eye Drop used/group**	**N**	**Minimum**	**25th percentile**	**Median**	**75th percentile**	**Maximum**	**Significantly Different Comparison between groups (*P* < 0.05)**
1) X-HA	10	2	3	3	6	7	(2)(3)
2) AMEED	10	3	4	6.5	7	7	(1)
3) Saline	10	4	6	7	7	7	(1)

**Table 3:** General descriptive statistics demonstrating results pertaining to the ulcerated area % of the total corneal surface observed per group investigated – median (interquartile range—IQR).

	**Ulcerated area % of the total corneal surface**					
**Treatment**	**Time (h)**					
	**0**	**12**	**24**	**36**	**48**	**72**
X-HA	35.01 (0.22)	26.17 (10.41)	8.88 (6.11)	2.73 (5.52)	0.00 (0.26)	0.00 (0.00)
AMEED	34.98 (0.66)	23.82 (12.55)	14.17 (8.55)	8.12 (9.99)	3.68 (8.25)	0.00 (4.93)
Saline (control)	35.20 (1.08)	26.91 (7.04)	14.49 (6.09)	9.95 (9.10)	6.30 (8.64)	1.44 (3.58)

**Figure 4:**
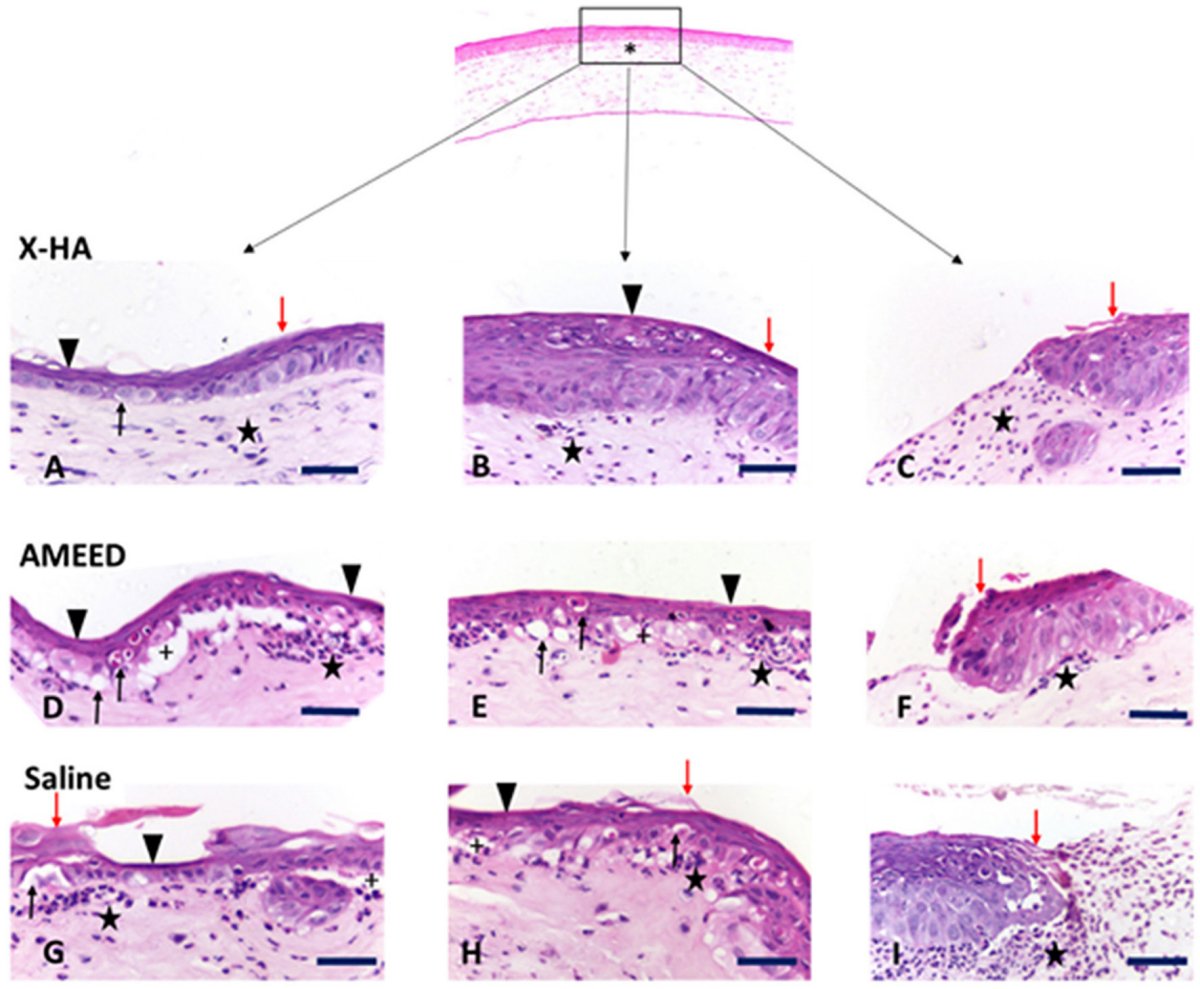
Photomicrographs demonstrating common histological changes encountered in the corneal samples from all groups. Top – asterisk (*) low magnification photomicrography showing the central corneal region (rectangle) where the epithelial defect was created and from where the samples were taken. Samples A, B, and C are from the X-HA group (animals #1, ID 35 OD; #11, ID E1 OD and #13, ID E3 OS, respectively); samples D, E, and F from the AMEED group (Animals # 15, ID E5 OD; # 12, ID E2 OD; # 4 ID 04 OD, respectively); and samples G, H, and I from the saline (control) group (animals #8, ID 9, OD; #9, ID 17 OS; #5, ID 05 OD, respectively). Selected examples of changes found in the X-HA group: Sample **(A)** shows moderate keratinization, epithelial attenuation (pit), mild epithelial edema, stromal fibroplasia, and mild neutrophil infiltration are evident. Sample **(B)** shows discrete keratinization, re-epithelialization of the epithelium with slight structural disorganization, mild stromal fibroplasia, mild stromal neutrophil infiltration, and mild neovascularization are present. Sample **(C)** shows discrete keratinization, mild stromal fibroplasia, mild neutrophil infiltration, and mild neovascularization. Selected examples of changes found in the AMEED group: Sample **(D)** shows epithelial attenuation, epithelial-stromal separation (subepithelial cleft formation), mild epithelial hydropic degeneration, and moderate subepithelial neutrophil infiltration; Sample **(E)** shows epithelial attenuation, mild-to-moderate epithelial hydropic degeneration, subepithelial cleft, discrete neovascularization, and mild subepithelial neutrophil infiltration; and Sample **(F)** shows corneal ulceration with keratinization at the epithelial margins, corneal edema and mild neutrophil infiltration in the stroma. Selected examples of changes found in the saline (control) group: Sample **(G)** shows epithelial attenuation, keratinization, mild epithelial hydropic degeneration, apparent subepithelial cleft formation, edema, and moderate subepithelial neutrophil infiltration. Sample **(H)** shows epithelial attenuation, keratinization, mild subepithelial neutrophil infiltration, mild epithelial hydropic degeneration, and apparent subepithelial cleft formation. Sample **(I)** shows corneal ulceration with initial signs of re-epithelization, edema, and marked subepithelial neutrophil infiltration. Scale bars – 50 μm.

**Figure 5:**
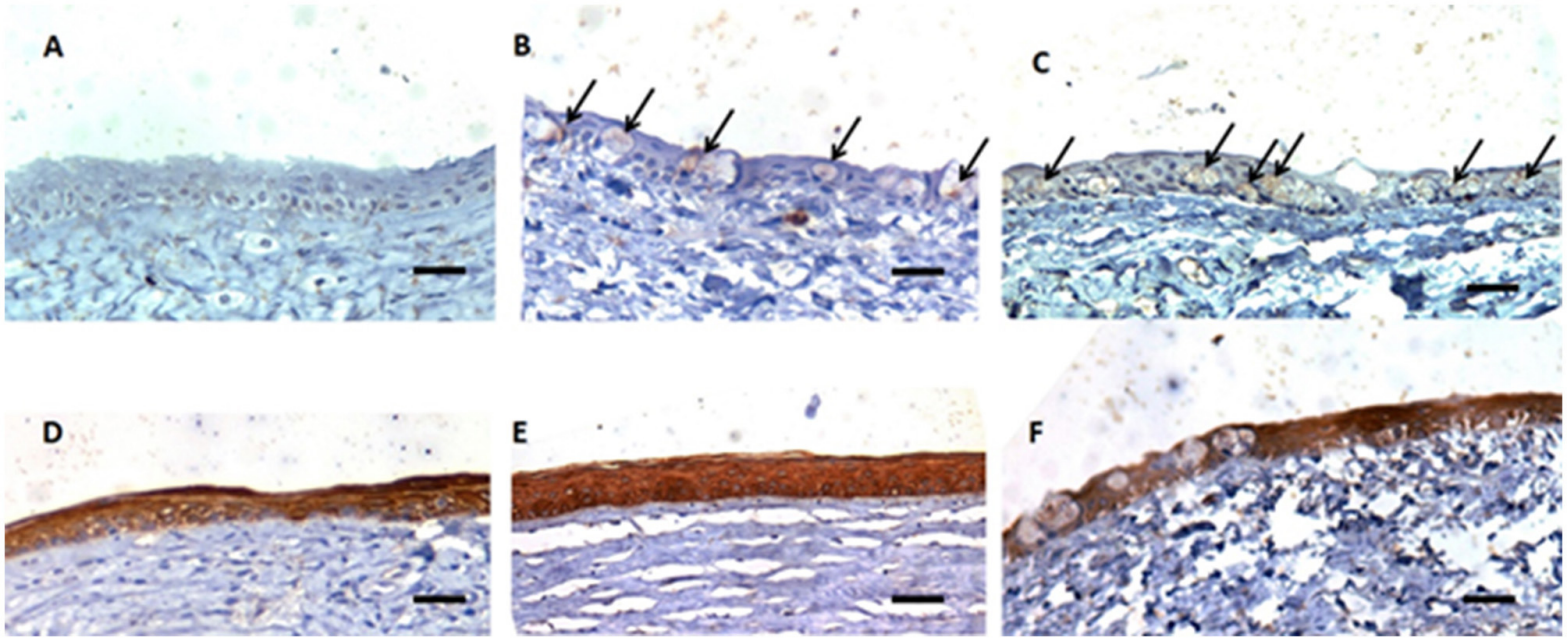
Representative photomicrographs demonstrating the immunohistochemistry reaction encountered in the corneal samples from all groups. Samples **(A)**: X-HA, **(B)**: AMEED, and **(C)**: Saline Groups, respectively, showed greater expression of cytoplasmic caspase 3 in the corneal epithelium in samples from the saline group **(C)**, compared to the treated groups **(A, B)** (arrows). Eye samples **(D)** (animal # 3, ID 42 OD): X-HA; **(E)**: AMEED (animal # 15, ID E5 OD); and **(F)** (animal # 10, ID 18 OS), belonging to the saline groups, respectively, showing equivalent labeling of cytokeratin in the corneal epithelium. Scale bars −50 μm.

The histopathologic analysis revealed significant differences between the groups. The following changes were observed: corneal epithelium attenuation, keratinization, inflammatory cell infiltrates (neutrophils), vascularization, hydropic degeneration of epithelial cells, and epithelial-stromal separation (subepithelial cleft formation). Histopathologic results are summarized in Table 4. Quantitative scores for changes such as keratinization, fibroplasia, and epithelium attenuation were homogeneously distributed among the individual samples from all different groups. However, the scores for inflammatory cell infiltrates were significantly lower (*P* = 0.027) in samples from the X-HA 1.5 (IQR 1)-treated group compared to those from the AMEED 2.0 (IQR 2.0)- and saline 3.0 (IQR 1.0)-treated groups (see Table 4 and Figure 4). In addition, inflammatory cell infiltrate tended to be found in a more subepithelial location in the AMEED and saline groups rather than being located in the anterior stroma in the X-HA and saline groups. Hydropic degeneration and epithelial-stromal separation (Figure 4) were features significantly (*P* = 0.0006) less common in samples from the X-HA-treated group, 0.0 (IQR 0.0), in comparison to samples from the AMEED-, 3.0 (IQR 1.0), and saline-, 2.0 (IQR 1.0), treated groups. Out of the 30 eyes, 12 samples from all groups showed complete and satisfactory re-epithelization. Non-re-epithelized areas were numerically more common in samples from the AMMED- (*n* = 5) and saline- (*n* = 5) treated groups than in samples from the X-HA-treated (*n* = 2) group. However, this change's attributed histopathologic intensity scores showed no significant differences between groups. Immunohistochemical analysis for cytokeratin and capase 3 antigens was identified for all samples. The corneal epithelium from all groups showed high and uniform cytoplasmic expression for cytokeratin, and no difference between the types of treatment was observed for labeling in terms of intensity or distribution in the corneal epithelium. Immunoexpression of caspase 3 was significantly more numerous in the epithelium cells (*P* = 0.00001) of samples from the AMEED and saline-treated groups (6.00 IQR 5.50 and 5.0 IQR 2.00) compared to those from the X-HA-treated group (0.00 IQR 0.00) (Figure 5).

**Table 4:** Absolute frequency (percentage) of the lesions encountered in the histopathologic analysis of the corneal epithelium and stroma from X-HA, saline (control), and AMEED groups.

**Group**	**Epithelium attenuation**	**Keratinization**	**Moderate subepithelial inflammatory cell infiltrate**	**Hydropic degeneration/epithelial-stromal separation**	**Corneal ulceration with incomplete re-epithelization**
X-HA	5 (50%)	8 (80%)	6 (60%)	0 (0%)	2
AMEED	8 (80%)	8 (80%)	8 (80%)	8 (80%)	5
Saline (control)	7 (70%)	9 (90%)	8 (80%)	2 (20%)	5

## 4 Discussion

The novelty of the present investigation compared to previous studies lies in the fact that it is the first to compare cross-linked hyaluronic acid (X-HA) and amniotic membrane extract (AME) in a controlled *in vivo* murine experimental model for corneal regeneration. Demonstrating evidence of the beneficial effects of X-HA, to our knowledge, this is the first study to bring additional comparative support from histopathologic and immunohistochemistry analyses.

Previous investigations corroborate our findings that topical treatment with X-HA is beneficial for corneal re-epithelization. However, these results and healing times are not directly comparable because other animal models were used (dogs, cats, rabbits, and human patients). For instance, an efficacy evaluation of X-HA for the treatment of corneal epithelial abrasion and standardized alkali burn injuries in rabbits demonstrated that the X-HA group reduced polymorphonuclear leukocytes during early healing and a smaller defect area after 5 days in the animal group receiving topical 1% X-HA compared to the control group (14). Additionally, formulations containing 0.16% or 0.32% concentrations of cross-linked sodium hyaluronate, taurine, vitamin B6, and vitamin B12 have also been shown to accelerate corneal wound healing in rabbits (15). Yu et al. (16) demonstrated improved corneal healing in dogs diagnosed with dry eye. Topically applied X-HA also significantly accelerated the healing of acute corneal stromal ulcers in dogs and cats compared with a linear hyaluronic acid topical solution (2). Moreover, the healing of epithelial defects created for photorefractive keratectomy (PRK) was accelerated with the use of X-HA compared to bandage lenses alone in human patients (17). It has been shown that an ophthalmic solution containing a combination of X-HA, coenzyme Q10, and vitamin E can protect the ocular surface of humans from potential damage on exposure to chlorinated water (18).

The significant difference in vascularization scores observed in the present study may be directly attributed to differences in neutrophil infiltration due to inflammatory stimuli. Clinical observation of vascularization in the eyes in these two groups also supports the histopathological observations. Infiltration of neutrophils is a known cause of corneal vascularization in mice (19, 20). In general, re-epithelization occurred with minimal opacity and vascularization in all groups compared to other corneal ulcer models in rats, such as alkaline-induced corneal injury (13).

Activated caspase 3 is associated with cell death in the apoptosis-inducing protease pathway as it cleaves key proteins involved in the cell repair process. Higher levels of cells labeled for cleaved caspase 3 cells in the corneal epithelium were observed in the saline and AMEED groups, suggesting that corneal samples from the X-HA-treated group were in more advanced healing stages at the time of the analysis. This pattern has been observed in the healing process of other forms of experimentally induced corneal epithelial disease, such as the scopolamine/exposure to an air draft dry eye model in mice (21), benzalkonium chloride-induced dry eye in rats (22), alkaline-induced corneal lesions in rats (23). Therefore, the findings can be interpreted as evidence of improved healing in the X-HA-treated group. The equivalent levels of immunostaining for cytokeratin observed in all three groups confirm that the cells being compared are indeed corneal epithelial cells since these cells normally express this marker in rats (24, 25).

The higher number of samples showing hydropic degeneration/subepithelial cleft formation in the AMEED group is concerning, suggesting that AMEED not only fails to accelerate corneal healing but may also demonstrate some mild toxicity. Epithelial-stromal detachment has been observed in rabbits exposed to toxic substances, such as mustard gas (26). The use of amniotic membrane eye drops (AMEED) involves a diverse range of manufacturing processes that lack standardization. Moreover, most human placental membrane products undergo devitalization following dehydration and irradiation. The impact of different preservation methods employed before AMEED production remains inadequately investigated, while unpreserved formulations exhibit limited shelf life post-production. Preserving AMEED effectively without compromising its efficacy poses challenges, particularly with regard to dehydration, irradiation, or cryopreservation techniques. Each distinct approach employed for processing and preserving AM may alter the structural and functional characteristics of the active biomolecules involved. Consequently, conducting bioassays on various AM products and derivatives is imperative to ensure standardized outcomes. Additionally, further investigation regarding the maintenance of microbiologic safety measures and routine donor screening procedures is still lacking (8).

According to a study conducted by He (27), immunostaining reveals abundant hyaluronic acid (HA) in the avascular stromal matrix of the AM. These results suggest that HA may be covalently linked with the heavy chains (HCs) of the inter-alpha-inhibitor (IalphaI) via a NaOH-sensitive bond. The HC-HA complex is likely to be one of the active components in AM responsible for its potential anti-inflammatory and anti-scarring effects in the cornea (27). However, it is still not clear whether HA in AM forms an HC-HA complex and if such a complex exerts any therapeutic action. The results from an investigation in a murine model of corneal abrasion demonstrated that AM and umbilical cord eye drops outperformed the control group by significantly expediting corneal epithelialization, thereby effectively promoting corneal epithelialization (5).

Furthermore, both amnion homogenate and transplanted AM were found to effectively promote corneal healing in a rabbit model, as demonstrated by Guo et al. (4). Additionally, the study suggested the need for further research on the usefulness of amnion homogenate. Our study, however, did not find a significant improvement in corneal healing in the AMEED-treated group. It is worth noting that, in the groups used in the study of Guo et al. (4), chloramphenicol 0.5% was employed, whereas our study did not include any antibiotics.

A single study in rats demonstrated that AMEED may help in early corneal stromal wound defect recovery (28). However, another study conducted by Lyons et al. (6) demonstrated no significant improvement in the healing rate when using AMEED for induced superficial corneal ulcers in horses. In their study, Lyons et al. (6) recommended further investigation to determine the potential benefits of using AMEED in infected or melting (malacic) equine corneal ulcers, as well as exploring different AMEED formulations. The present study also corroborates the results of Silveira et al. (7), who focused on re-epithelization and showed that AMEED did not decrease corneal re-epithelialization time in cats with experimentally superficial corneal ulcers. However, the latter group of authors mentioned in their investigation that topical antibiotics and a topical mydriatic agent (1% atropine) were applied 5 min before the administration of AMEED.

The focus of the present study was to investigate corneal re-epithelization in an established model of superficial corneal ulceration. Because of this fact, there was a conscious effort to create a superficial standardized superficial lesion of the cornea in all steps of the procedure while demarcating the epithelium with the trephine or while polishing the ulcer bed with a fine diamond burr. However, it is conceivable that some adjacent stromal layer was also removed during the process, which is a recognized limitation of this method, as faced by other authors, including Reid et al. (29), Hutcheon et al. (30), Portela et al. (10), Nagai et al. (31), and Katakami et al. (32). Nevertheless, the procedure was repeatable and was performed in the same way for all eyes (as described above). Therefore, the possibility of stromal removal and its eventual quantity were equally distributed among all eyes from all groups, not affecting the analysis and conclusions.

Although the topical use of X-HA is not currently widespread in human ophthalmology, there are X-HA acid eye drop formulations in different concentrations for human use, such as 0.10% (VisuXL^®^, VISUfarma, Amsterdam, The Netherlands) and 0.75% (KIO-201, Kiora Pharmaceuticals, Encinitas, CA, USA) (18, 33). In contrast, veterinary X-HA formulations that are widely used include 40% Ocunovis Procare^®^ SentrX, Salt Lake City, UT, USA) and 0.75% (Oculenis Biohance^®^, SentrX, Salt Lake City, UT, USA) concentrations. Conversely, there are no commercially available amniotic membrane extract eye drops for human use. Evidence derived from veterinary drugs in animals exemplifies an underutilized resource that may well serve as a link between information gained from animal models and human clinical trials (34). For example, cyclosporine is widely used to treat keratoconjunctivitis sicca (KCS) and was only identified as a potential therapeutic for human use after veterinary ophthalmologists reported its efficacy in treating dogs with naturally occurring KCS (34, 35).

## 5 Conclusion

X-HA eye drops improved corneal epithelialization, as reflected in decreased re-epithelization time and a smaller median ulcerated area. This improvement was consistent across all evaluation times compared to eyes from the AMEED- and saline- (control) treated groups in rats with experimental superficial corneal ulcers. AMEED did not decrease corneal re-epithelialization time and demonstrated mild signs of corneal epithelium toxicity in samples analyzed under light microscopy.

## Data availability statement

The original contributions presented in the study are included in the article/supplementary material, further inquiries can be directed to the corresponding author.

## Ethics statement

The study was approved by the Research Ethics Committee of Faculdades Pequeno Príncipe (https://faculdadespequenoprincipe.edu.br). Address: Avenida Iguaçu 333—Block 2–1st floor, Rebouças, CEP: 80230-020, Curitiba/Paraná/Brasil and was conducted in accordance with the local legislation and institutional requirements.

## Author contributions

LG: Investigation, Writing – original draft, Methodology, Formal analysis, Visualization. CS: Methodology, Writing – review & editing, Investigation, Conceptualization, Data curation, Formal analysis, Funding acquisition, Project administration, Resources, Software, Supervision, Validation, Visualization. MV: Writing – review & editing, Methodology, Visualization, Formal analysis, Investigation. EP: Investigation, Writing – review & editing, Visualization. TG: Investigation, Writing – review & editing, Methodology. EF: Data curation, Investigation, Writing – review & editing, Methodology, Visualization. FM-F: Conceptualization, Formal analysis, Investigation, Methodology, Project administration, Resources, Software, Supervision, Writing – review & editing. JM: Methodology, Investigation, Writing – review & editing.
